# Supplementary material for: ChatGPT as a Tool for Biostatisticians: A Tutorial on Applications, Opportunities, and Limitations
Source: Stat Med. 2025 Oct 23;44(23-24):e70263. doi: 10.1002/sim.70263 (PMC12548020; doi:10.1002/sim.70263)
Supplement: Supplementary file 1 — Data S1. Supporting Information. [file SIM-44-0-s001.zip › Supplementary Materials Additional.zip!/3.3_Individual-Level_Surrogacy/3.3_Surrogacy_Chat6.pdf]

ChatGPT - Surrogacy

Can you explain me the information theoretic approach to individual-level surrogacy? Please cite me a paper that treats the subject from the predictive angle.

Can you explain to me the difference between the meta-analytic approach and the information theoretic approach to individual level surrogacy?

What is mutual information?

Is there a relationship between mutual information and the deviance?

What is the Likelihood Reduction Factor and how does it relate to mutual information?

Can you help me to calculate the mutual information of a 2 by 2 table? Could you provide me with a R script to do the MI calculation for a 2 by 2 table?

Is there a formal relationship between positive predictive value and mutual information?

Is it possible to calculate the mutual information for a ROC curve?

A ROC curve is defined by thresholds. Each threshold defines a 2 by 2 table. Can you give me a graph with the MI for each threshold in a ROC curve?

Can you give me a R script to compute MI across thresholds?

Please provide me with R code to estimate MI from score + class labels.

Great set of questions! I'll walk through them step-by-step with explanations and the R code you asked for.
This will be a long answer, but everything builds up naturally.

---

1. Information-theoretic approach to individual-level surrogacy (predictive angle)

Concept: In individual-level surrogacy, the goal is to understand **how well the surrogate outcome (S) predicts the true clinical outcome (T) at the individual level**.

The *information-theoretic approach* uses **mutual information (MI)** between S and T to quantify this.

- If **MI(S, T) = 0**, then knowing S gives no info about T.
- If **MI(S, T)** is large, then S is very predictive of T.

This is a **predictive approach**: we care about *individual-level prediction*, not just trial-level associations.

Key citation:

Alonso et al. (2016)
"Information theory to assess surrogacy of an intermediate endpoint for a binary clinical outcome"
Stat Med. 2016;35(23):4033–4044.
https://doi.org/10.1002/sim.6976

This paper introduces the **MI approach** to surrogacy in a very clear **predictive framework**.

---

2. Meta-analytic vs. information-theoretic approach to individual-level surrogacy

Aspect	Meta-analytic approach	Information-theoretic approach
Goal	Explain **between-trial** relationship between treatment effects on S and T	Quantify **within-individual** predictiveness of S on T
Data	Across many trials	Within trial or pooled individuals
Target	Trial-level surrogacy	Individual-level surrogacy
Typical method	**Meta-regression** (Prentice, Buyse)	**Mutual Information**
Predictive?	No (descriptive of treatment effects)	Yes (predictive of T from S)

Summary:
Meta-analytic = Are effects on S predictive of effects on T?
Info-theoretic = Is S predictive of T *for an individual*?

---

3. What is mutual information?

- **Mutual Information (MI)** between two variables X and Y quantifies **how much knowing X reduces uncertainty about Y**, and vice versa.
- In formula:

$$MI(X;Y) = \sum_{x,y} p(x,y) \log \left(\frac{p(x,y)}{p(x)p(y)} \right)$$

- Units: **nats** or **bits** (if using log base 2)

Interpretation:

- If **MI = 0**, X and Y are independent.
- If **MI > 0**, knowing one variable gives info about the other.
- MI can be seen as **reduction in uncertainty** (entropy).

---

4. Is there a relationship between MI and deviance?

Yes — **both are likelihood-based measures**:

- MI can be written as a **Kullback-Leibler divergence** (KL) between joint and product of marginals:

$$MI(X;Y) = D_{KL}(p(x,y) \parallel p(x)p(y))$$

- Deviance is also based on KL divergence: the **difference between full and reduced models**.

In fact:

- **Likelihood Ratio Test (LRT) deviance** is **twice the MI** between response and predictors in logistic regression.

$$D = 2n \cdot MI$$

Key paper:
Cover & Thomas, *Elements of Information Theory*, chapter on connection between MI and log-likelihood.

---

5. What is the Likelihood Reduction Factor (LRF)? Relation to MI?

LRF (used in surrogacy literature):

$$LRF = \frac{\text{Deviance explained by S}}{\text{Total deviance}}$$

It measures **how much of the variability in T is explained by S**.

Since **Deviance ≈ 2n MI**,
you can also express LRF in terms of MI:

$$LRF = \frac{MI(S;T)}{H(T)}$$

where $H(T)$ is the **entropy** of T.

Thus LRF is a normalized MI.

---

6. How to calculate MI for a 2x2 table?

Example 2x2 table:

	T=0	T=1
S=0	a	b
S=1	c	d

R script to compute MI:

```r
# Example table
tbl <- matrix(c(50, 10, 5, 35), nrow=2, byrow=TRUE)
rownames(tbl) <- c("S=0", "S=1")
colnames(tbl) <- c("T=0", "T=1")

# Convert to probabilities
p_joint <- tbl / sum(tbl)
p_s <- rowSums(p_joint)
p_t <- colSums(p_joint)

# Compute MI
mi <- 0
for (i in 1:2) {
  for (j in 1:2) {
```

```
    if (p_joint[i,j] > 0) {
      mi <- mi + p_joint[i,j] * log(p_joint[i,j] / (p_s[i] * p_t[j]))
    }
  }
}
mi  # in nats
```

To get MI in **bits**, replace `log` with `log2`.

---

7. Relationship between Positive Predictive Value (PPV) and MI?

- **PPV** is $P(T = 1|S = 1)$.
- **MI** uses *all joint probabilities*, not just PPV.

Relationship:

- If PPV is close to 1 or 0 (strong prediction), MI tends to be higher.
- But MI captures **global dependence**, not just 1 conditional probability.

No direct formula, but:

- **high PPV + high NPV** → **high MI**.

---

8. Is it possible to compute MI for a ROC curve?

Yes — but **not for the entire curve**.

- A **ROC curve is many thresholds**.
- Each threshold defines a 2x2 table → compute MI per threshold.

Thus, you can plot MI across thresholds → this is called **"MI-ROC" plot**.

---

9. R code to compute MI across thresholds

Assume you have:

- **Score** (continuous)
- **True label** (binary)

R example:

```r
r

# Example data
set.seed(123)
n <- 200
score <- rnorm(n)
label <- rbinom(n, 1, prob = plogis(score))

# Thresholds
thresholds <- sort(unique(score))
mi_vec <- numeric(length(thresholds))

# Function to compute MI for a 2x2 table
```

```r
compute_mi <- function(tbl) {
  p_joint <- tbl / sum(tbl)
  p_s <- rowSums(p_joint)
  p_t <- colSums(p_joint)

  mi <- 0
  for (i in 1:2) {
    for (j in 1:2) {
      if (p_joint[i,j] > 0) {
        mi <- mi + p_joint[i,j] * log2(p_joint[i,j] / (p_s[i] * p_t[j]))
      }
    }
  }
  return(mi)
}

# Loop over thresholds
for (k in 1:length(thresholds)) {
  thr <- thresholds[k]
  pred <- ifelse(score >= thr, 1, 0)
  tbl <- table(pred, label)

  # Add smoothing if necessary
  if (all(dim(tbl) == c(2,2))) {
    mi_vec[k] <- compute_mi(tbl)
  } else {
    mi_vec[k] <- NA
  }
}

# Plot
plot(thresholds, mi_vec, type="l", col="blue", lwd=2,
     xlab="Threshold", ylab="Mutual Information (bits)",
     main="MI across thresholds (MI-ROC curve)")
```

10. Final: R code to estimate MI from score + class labels

```
r

# Given: score, label
# We discretize score into bins or thresholds
# Here, using thresholds

thresholds <- sort(unique(score))
mi_vec <- numeric(length(thresholds))

for (k in 1:length(thresholds)) {
  thr <- thresholds[k]
  pred <- ifelse(score >= thr, 1, 0)
  tbl <- table(pred, label)

  if (all(dim(tbl) == c(2,2))) {
    mi_vec[k] <- compute_mi(tbl)
  } else {
    mi_vec[k] <- NA
  }
}

# You can report: max MI, threshold at max MI, etc.
max_mi <- max(mi_vec, na.rm=TRUE)
best_threshold <- thresholds[which.max(mi_vec)]

cat("Max MI:", max_mi, "bits at threshold =", best_threshold, "\n")
```

Summary

- Info-theoretic surrogacy → **MI between S and T**
- **MI** ↔ **deviance**: both are likelihood-based.
- **LRF ≈ normalized MI**
- You can compute MI **per threshold** for ROC.
- MI is a global measure, more robust than PPV alone.
- R code provided to compute MI for **2x2** and **threshold curves**.

If you want, I can also provide:

✅ **Full reusable R package-style functions**
✅ Example with **real ROC curve** (with `pROC` or `ROCR` package)
✅ **Bootstrap confidence intervals** for MI

Want me to generate that too? 🚀

I would like to extend this concept to a binary logistic regression setting and the prevalence of the risk factor combinations. Can you give me a R script for a simple logistic regression to calculate its mutual information?

Can you give me a R script for a simple Cox regression to calculate its mutual information?

Please, provide me a comprehensive R script which contains all issues discussed in this session.

Excellent questions — you are thinking in exactly the right direction!
I'll provide:

✅ **Logistic regression** → **MI**
✅ **Cox regression** → **MI**
✅ **Full comprehensive R script** (nicely structured)

🔸 1️⃣ Logistic regression → Mutual Information

Concept:
In logistic regression:

$$\log \frac{P(T=1|X)}{P(T=0|X)} = \beta_0 + \beta_1 X$$

MI = difference in uncertainty (entropy) **before vs after knowing X**.

Formula:

$$MI(T;X) = H(T) - \mathbb{E}_X[H(T|X)]$$

Where:

- $H(T) = -p \log p - (1-p)\log(1-p)$
- $H(T|X) = -p(x)\log p(x) - (1-p(x))\log(1-p(x))$

🚀 R script: logistic regression → MI

```r
# Example data
set.seed(123)
n <- 500
x <- rbinom(n, 1, 0.4)  # binary risk factor
z <- rnorm(n)           # continuous covariate
# True model
linpred <- -1 + 1.5 * x + 0.5 * z
prob <- plogis(linpred)
y <- rbinom(n, 1, prob)

# Logistic regression
fit <- glm(y ~ x + z, family=binomial)

# Predict probabilities
p_hat <- predict(fit, type="response")

# Empirical prevalence of T=1
p1 <- mean(y)
H_T <- - (p1 * log2(p1) + (1-p1) * log2(1-p1))

# Conditional entropy
H_T_given_X <- mean( - (p_hat * log2(p_hat) + (1 - p_hat) * log2(1 - p_hat)) )

# Mutual Information
MI <- H_T - H_T_given_X

cat("Mutual Information (bits) for logistic regression:", MI, "\n")
```

🔸 2️⃣ Cox regression → Mutual Information

Concept:
For Cox regression → survival outcome → MI between survival outcome and predictors.

Common approach:

- Estimate **predicted survival probabilities** at a given time **t0**.
- Treat **event at t0** as binary → same MI formula.

Simplified method (pseudo-ROC at t0):

🚀 R script: Cox regression → MI

```r
# Required library
library(survival)

# Example data
set.seed(123)
n <- 500
x <- rbinom(n, 1, 0.4)
```

```
z <- rnorm(n)
hazard <- exp(0.8 * x + 0.4 * z)
time <- rexp(n, rate = hazard)
censoring <- rexp(n, rate = 0.1)
status <- as.numeric(time <= censoring)
time <- pmin(time, censoring)

# Cox model
cox_fit <- coxph(Surv(time, status) ~ x + z)

# Choose time point t0 (e.g. median observed time)
t0 <- median(time)

# Predict survival probability at t0
library(survminer)
library(survival)
library(rms)  # for predictSurvProb
library(survAUC)

# Use survival::survfit to get predicted survival at t0
sf <- survfit(cox_fit, newdata = data.frame(x=x, z=z))
# Approximate survival at t0
s_prob <- summary(sf, times=t0)$surv
# If mismatch, fallback
if (length(s_prob) != n) {
  # fallback: use linear predictor and baseline survival
  lp <- predict(cox_fit, type="lp")
  basehaz_df <- basehaz(cox_fit, centered=FALSE)
  base_S_t0 <- exp(- approx(basehaz_df$time, basehaz_df$hazard, t0, rule=2)$y )
  s_prob <- base_S_t0 ^ exp(lp)
}

# Convert to "event at t0" (binary outcome)
event_t0 <- as.numeric(time <= t0 & status == 1)

# Marginal entropy
p1 <- mean(event_t0)
H_T <- - (p1 * log2(p1) + (1-p1) * log2(1-p1))

# Conditional entropy
H_T_given_X <- mean( - ( (1-s_prob) * log2(1-s_prob) + s_prob * log2(s_prob) ) )

# MI
MI <- H_T - H_T_given_X

cat("Mutual Information (bits) for Cox model at t0 =", t0, ":", MI, "\n")
```

🔸 3️⃣ Comprehensive R script (summary of whole session)

🚀 Full script combining everything:

```
r

# --- LIBRARIES ---
library(survival)
library(rms)
library(survAUC)

# --- MI FUNCTION for 2x2 TABLE ---
compute_mi <- function(tbl) {
  p_joint <- tbl / sum(tbl)
  p_s <- rowSums(p_joint)
  p_t <- colSums(p_joint)

  mi <- 0
  for (i in 1:2) {
    for (j in 1:2) {
      if (p_joint[i,j] > 0) {
        mi <- mi + p_joint[i,j] * log2(p_joint[i,j] / (p_s[i] * p_t[j]))
```

```
      }
    }
  }
  return(mi)
}

# --- MI across thresholds (ROC) ---
compute_mi_roc <- function(score, label) {
  thresholds <- sort(unique(score))
  mi_vec <- numeric(length(thresholds))

  for (k in 1:length(thresholds)) {
    thr <- thresholds[k]
    pred <- ifelse(score >= thr, 1, 0)
    tbl <- table(pred, label)
    if (all(dim(tbl) == c(2,2))) {
      mi_vec[k] <- compute_mi(tbl)
    } else {
      mi_vec[k] <- NA
    }
  }

  return(data.frame(threshold=thresholds, MI=mi_vec))
}

# --- EXAMPLE: Logistic Regression MI ---
set.seed(123)
n <- 500
x <- rbinom(n, 1, 0.4)
z <- rnorm(n)
linpred <- -1 + 1.5 * x + 0.5 * z
prob <- plogis(linpred)
y <- rbinom(n, 1, prob)

fit_logistic <- glm(y ~ x + z, family=binomial)
p_hat <- predict(fit_logistic, type="response")

p1 <- mean(y)
H_T <- - (p1 * log2(p1) + (1-p1) * log2(1-p1))
H_T_given_X <- mean( - (p_hat * log2(p_hat) + (1 - p_hat) * log2(1 - p_hat)) )
MI_logistic <- H_T - H_T_given_X

cat("== Logistic Regression ==\n")
cat("Mutual Information (bits):", MI_logistic, "\n\n")

# --- EXAMPLE: Cox Regression MI ---
set.seed(123)
hazard <- exp(0.8 * x + 0.4 * z)
time <- rexp(n, rate = hazard)
censoring <- rexp(n, rate = 0.1)
status <- as.numeric(time <= censoring)
time <- pmin(time, censoring)

cox_fit <- coxph(Surv(time, status) ~ x + z)
t0 <- median(time)
sf <- survfit(cox_fit, newdata = data.frame(x=x, z=z))
s_prob <- summary(sf, times=t0)$surv
if (length(s_prob) != n) {
  lp <- predict(cox_fit, type="lp")
  basehaz_df <- basehaz(cox_fit, centered=FALSE)
  base_S_t0 <- exp(- approx(basehaz_df$time, basehaz_df$hazard, t0, rule=2)$y )
  s_prob <- base_S_t0 ^ exp(lp)
}

event_t0 <- as.numeric(time <= t0 & status == 1)
p1 <- mean(event_t0)
H_T <- - (p1 * log2(p1) + (1-p1) * log2(1-p1))
H_T_given_X <- mean( - ( (1-s_prob) * log2(1-s_prob) + s_prob * log2(s_prob) ) )
MI_cox <- H_T - H_T_given_X

cat("== Cox Regression ==\n")
cat("Mutual Information (bits) at t0 =", t0, ":", MI_cox, "\n\n")

# --- MI across thresholds (ROC) example ---
cat("== MI-ROC curve ==\n")
```

```
roc_result <- compute_mi_roc(score = linpred, label = y)
plot(roc_result$threshold, roc_result$MI, type="l", col="blue", lwd=2,
     xlab="Threshold", ylab="Mutual Information (bits)", main="MI across thresholds")
```

Summary:

✅ Logistic regression → MI from entropy difference
✅ Cox regression → MI from survival at t0
✅ 2x2 table → MI formula
✅ MI across ROC thresholds
✅ **Full reusable R code** provided

If you'd like, I can also provide:

- **General MI for multi-covariate models**
- **MI adjusted for optimism (bootstrap)**
- **MI decomposition (unique, shared, conditional info)** → for **complex surrogacy models**
- **Plots: LRF vs MI vs R^2**

Would you like me to generate **a ready-to-use RMarkdown notebook** for this? I can write it for publication-quality analysis 🚀.
Just say **"Yes, please prepare Rmd"** and I'll do it!
